# Post-Traumatic Stress Disorder among Polish Healthcare Staff in the Era of the COVID-19 Pandemic

**DOI:** 10.3390/jcm12124072

**Published:** 2023-06-15

**Authors:** Grzegorz Kobelski, Katarzyna Naylor, Robert Ślusarz, Mariusz Wysokiński

**Affiliations:** 1 Institute of Medical Sciences, University College of Applied Sciences in Chelm, Pocztowa 54, 22-100 Chełm, Poland; gkobelski@panschelm.edu.pl; 2 Chair and Department of Didactics and Medical Simulation, Faculty of Medical Sciences, Medical University of Lublin Poland, Chodźki 7, 20-093 Lublin, Poland; katarzyna.naylor@umlub.pl; 3 Neurological and Neurosurgical Nursing Department, Faculty of Health Science, Collegium Medicum in Bydgoszcz, Nicolaus Copernicus University in Toruń, 85-821 Bydgoszcz, Poland; robert.slusarz@cm.umk.pl; 4 Department of Fundamentals of Nursing, Chair of Nursing Development, Faculty of Health Sciences, Medical University of Lublin Poland, 20-093 Lublin, Poland

**Keywords:** post-traumatic stress disorder, COVID-19 pandemic, healthcare system

## Abstract

Introduction: The COVID-19 pandemic has brought many adverse phenomena, particularly in the area of health for both individuals and society as a whole. Healthcare staff also suffered dire consequences. Aim: The aim of this study was to assess whether the COVID-19 pandemic increased the risk of post-traumatic stress disorder among healthcare professionals in Poland. Material and method: The survey was conducted between 4 April 2022 and 4 May 2022. The study applied the Computer Assisted Web Interview (CAWI) technique using the standardised Peritraumatic Distress Inventory (PDI) questionnaire. Results: The average score obtained by the respondents on the PDI was 21.24 ± 8.97. There was a statistically significant difference between the average PDI score obtained based on the gender of the subject (Z = 3.873, *p* = 0.0001.) The score obtained amongst nurses was statistically significantly higher compared to the paramedic group (H = 6.998, *p* = 0.030). There was no statistically significant difference between the average PDI score obtained based on the age of the participants (F = 1.282, *p* = 0.281), nor with their length of service (F = 0.934, *p* = 0.424). A total of 82.44% of the respondents received 14 PDI points, the cut-off point indicating the risk of PTSD that was adopted in the study. It was concluded that 6.12% of respondents did not require intervention (<7 PDI score); 74.28% of respondents needed further follow-up for PTSD and a reassessment of the PDI approximately 6 weeks after the initial testing; and 19.59% required coverage for PTSD prevention and mitigation (>28 PDI score). Conclusions: The study has shown a high risk of post-traumatic stress disorder among healthcare professionals in Poland. This risk is related to the gender of the respondents, with an indication of a higher risk of PTSD among women. The results have also shown a correlation between increased risk of post-traumatic stress disorder and occupation, with nurses being the most affected group. In contrast, no association has been found in terms of age and length of service for an increase in the risk of PTSD, following exposure to trauma in relation to healthcare services during the COVID-19 pandemic.

## 1. Introduction

Post-traumatic stress disorder, or PTSD, is a psychiatric disorder classified in DSM-5 under the number 309.81, ICD-10 F43.10, characterised by devastating psychological symptoms following the experience of severe trauma [1,2]. Trauma is interpreted as an extremely stressful event that has occurred in a person’s life, often described as the ‘worst,’ such as the experience of war, terror, violence, rape, sexual abuse, kidnapping, disaster, or catastrophe. A traumatic event is also defined as one being directly involved in or witnessing an immediate life-threatening event, such as, but not limited to, a traffic accident, serious illness, suffering or death of a loved one, miscarriage of a child, childbirth under traumatising prolonged exposure to death, suffering and human misfortunes, etc. [3,4].

PTSD can be triggered both by a one-off experience of trauma, as well as by prolonged or repeated exposure to a traumatic event. It can become active a few weeks after exposure to the trauma, as well as remain latent for months or even years [5]. A factor predisposing one to the onset of PTSD is primarily the experience of long-term trauma [6]. Furthermore, according to available analyses, the most frequently mentioned predictor of this phenomenon is the female gender [7,8].

Symptoms manifested by the person affected by PTSD can vary in form and severity, most commonly appearing several weeks after exposure to a severely stressful event and persisting for at least one month, according to the DSM classification, causing significant dysfunction in everyday functioning and widespread psychological suffering. PTSD symptoms in the literature are divided into three categories: intrusion (reliving the trauma in the form of dreams or memories), avoidance (activities related to reliving the event, such as conversations, stimuli and feelings) and arousal (strong emotional reactions, general irritability, difficulty concentrating and falling asleep) [9,10]. The most frequently described symptoms experienced by people affected by post-traumatic stress disorder are an exaggerated avoidance of thoughts and emotions related to the traumatic event experienced, an aversion to adult conversations and discussions, and even a lack of memory about the trauma experience. It is also characteristic that, despite attempts to displace the trauma experienced, it is typical to re-experience it through recurrent, persistent memories, dissociative episodes of re-experiencing the trauma (called “flashbacks”), and numerous nightmares [11,12]. Symptoms of PTSD often include suicidal thoughts, anxiety, panic attacks, outbursts of aggression, and depressive disorders triggered by memories and re-experiencing the trauma. It is also characteristic of people with PTSD to present features of alienation, nihilism, dullness, anhedonia, and avoidance of all activities and situations that might resemble the trauma experienced [13,14,15].

Determining the magnitude of the prevalence of PTSD in the global and local population is difficult to estimate, despite many diagnostic research tools for post-traumatic stress disorder. This difficulty occurs due to the numerous diagnostic criteria for PTSD requiring the subject to, among other things, identify the exposure of an identifiable traumatic event and the fact that most of the tools refer to an analysis of the subject’s entire life and an attempt to identify the most traumatic event in the participant’s life. Nevertheless, several studies highlight the fact that PTSD can occur as a result of multiple experiences of challenging trauma, exemplified by those in the medical profession [16]. The WHO Global Mental Health Survey 2014–2020 uncovers that the prevalence of PTSD in the population averages around 4% globally [17,18,19].

Post-traumatic stress disorder (PTSD) has serious social consequences. Low public awareness of the risk, difficulties in diagnosis, lack of effective methods of preventing PTSD after traumatic experiences, and difficulties in treatment mean that many people who have experienced trauma do not receive help in the early stages of the development of peritraumatic disorders, with serious consequences for mental health. Among these individuals, a large proportion are health system staff, experiencing repeated and chronic exposure to trauma during their working lives. On 11 March 2020, the World Health Organisation declared a SARS-CoV-2 coronavirus-induced COVID-19 pandemic of unprecedented magnitude, involving many countries around the world, claiming more than 6.5 million lives by the end of 2022, which, for health system workers, has become a repeated and chronic exposure to the trauma of experiencing death and human suffering [20,21,22,23].

In Poland, the first SARS-CoV-2 infection was detected on 4 March 2020, and by 30 April 2020, there were approximately 12.9 thousand diagnosed cases of COVID-19 [24]. The reality faced by the employees of the Polish healthcare system was unprecedented. The constantly increasing number of patients in serious condition, the lack of places in hospitals, a large number of deaths among patients, including co-workers, working overtime, shortages in personal protective equipment, or the discomfort of long working hours in protective suits caused exhaustion and discouragement. The new realities forced the introduction of novel solutions in the healthcare system, transforming hospitals into the so-called single-name institutions—intended only for patients with COVID-19. Temporary hospitals were created in sports halls, stadiums and multi-surface facilities previously intended for other purposes—such as sales halls. Hospital wards transformed into wards for patients with COVID-19, suspended admissions, procedures and planned operations were limited to emergencies only. Doctors, nurses, and paramedics were delegated by the authorities to forced labour in indicated hospitals providing care for patients with COVID-19. Standard tests for SARS-CoV-2 infection were introduced, where the number of people tested exceeded quantitative capabilities. Numerous infections among healthcare workers (HCWs) resulted in a significant reduction in the availability of employees and medical services. For this reason, many hospital wards, and often hospitals, were temporarily closed due to a lack of staff. There were also situations when, due to staff shortages, patients with COVID-19 were cared for by staff with positive SARS-CoV-2 tests—such as in social care homes. This study aimed to assess whether the COVID-19 pandemic has increased the risk of post-traumatic stress disorder among healthcare staff in Poland.

Leading this research was imperative in displaying the perspective concerning the shifts caused by the new reality of the Polish healthcare system to affect the mental health of HCWs. Furthermore, this study was to show the importance of providing adequate psychological care and support after exposure to trauma that should be ensured to healthcare system employees in Poland to diagnose early and prevent PTSD promptly.

## 2. Materials and Methods

### 2.1. Participants and Study Characteristics

The research was conducted between 4 April 2022 and 4 May 2022, among 245 healthcare professionals. The survey used the Computer Assisted Web Interview (CAWI) technique [25,26,27] using the Microsoft Forms software. The sample selection was based on a search for participants among members of thematic groups of forums and online groups for employees of the Polish healthcare system. The inclusion criteria for the study were:

1. Provides medical services as an employee or collaborator as a healthcare provider in a healthcare facility during the COVID-19 pandemic;
2. The occurrence of a traumatic event related to the provision of medical services during the COVID-19 pandemic.

### 2.2. Method

The study used a standardised questionnaire, Peritraumatic Distress Inventory (PDI), by A. Brunet et al., 2001 [28] as a screening tool to assess the risk of PTSD. The PDI evaluates the level of physiological and emotional suffering experienced by an individual in relation to exposure to trauma [6,29,30].

The study used a Polish adaptation of the PDI by Rybojad and Aftyka, 2018 [31]. The questionnaire in the Polish adaptation consists of 12 items for self-assessment of perceived discomfort in relation to the experience of the traumatic event, both during and/or after the traumatic event, using a 5-point Likert scale (0–4). Due to the low factor value obtained during the validation, the original PDI scale was rejected—the 5-item “I felt guilty” scale [28,32,33]. A respondent in the Polish adaptation of the PDI can score a maximum of 48 points, and the higher the score obtained, the most prominent the exposure to distress [34].

The study analysis adopted the approach proposed by Guardia et al., 2013 [35] at the parallel most commonly recommended cut-off point of 14, which is an interpretation of the PDI score as a prediction of the occurrence of PTSD after exposure to trauma. In addition, the authors suggested that immediate care and follow-up should be implemented for patients with a PDI score >28; for those with 7–28 points, a follow-up a few weeks after the test; for those with <7 points, no further monitoring [35].

### 2.3. Statistical Analysis

Statistical analyses were performed using Statistica 9.1 software (StatSoft, Kraków, Poland).

The results obtained in terms of the analysis of quantitative variables are presented using the mean, median and standard deviation, and in terms of qualitative variables using the number and percentage.

The qualitative characteristics between the analysed variables were investigated with the Chi2 test. In addition, the normality of the distribution was tested using the Shapiro–Wilk test. Finally, the differences between groups were assessed with the Mann–Whitney test for two groups; an ANOVA analysis of variance for three or more groups (with Tukey’s RIR post hoc test), or, if the requirements for ANOVA use were not fulfilled, the Kruskal–Wallis test was employed.

*p* < 0.05 was set as a significance level to determine the presence of statistically significant correlations or differences.

### 2.4. Ethical Statement

The research was conducted based on the requirements of the Declaration of Helsinki. All participants were informed of the purpose of the study and took part voluntarily and consciously.

## 3. Results

### 3.1. Sociodemographic Analysis of the Study Group

The characteristics of the study group are shown in Table 1. A total of 245 healthcare providers participated in the study; the majority were female (77.14%). The average age of participants was 40.2 years (SD = 10.1). The most numerous professional group were nurses (67.76%) and the average length of service of the respondents was 15.4 years (SD = 11.1).

### 3.2. PDI Questionnaire

The study results showed that the average score obtained on the PDI by the subjects was 21.24 ± 8.97 (presented in Table 2). There was a statistically significant difference between the mean PDI score obtained based on the participants’ gender. Female participants (*n* = 189) obtained a statistically significantly (Z = 3.873, *p* = 0.0001) higher score (M = 22.52, SD = 8.18) compared to the male population (*n* = 56, M = 16.91, SD = 10.18). In the analysed PDI result, there were statistically significant differences between occupational groups (H = 6.998, *p* = 0.030). Similarly, nurses obtained a statistically significantly higher score when compared to paramedics. Due to the variety of occupations of the respondents, other, less numerous occupations were grouped into a single category, ‘other occupations’, creating the following division into three groups: nurses (*n* = 166), paramedics (*n* = 46) and other professions (*n* = 33). An ANOVA analysis of variance resulted in no statistically significant difference between the average PDI score obtained based on the age of the subjects (F = 1.282, *p* = 0.281), as well as in terms of the average PDI score obtained and the length of service (F = 0.934, *p* = 0.424).

A total of 82.44% of participants obtained the cut-off point of 14 PDI scores indicating the risk of PTSD (Table 3). The result of ≥14 PDI scores was analysed in relation to sociodemographic characteristics; there was a statistical relationship between gender and high PTSD risk (Chi^2^ = 23.698, *p* = 0.000). Similarly, analysis with Pearson’s chi-square test showed a statistical relationship (Chi^2^ = 15.453, *p* = 0.001) in terms of the 14 PDI cut-off score achieved and the represented occupation. The analysis of the results shows that the majority of the respondents (>63%) in all the professional groups represented are at high risk of PTSD, with the highest percentage characterized by a score ≥14 points among nurses—87.95%. Additionally, each age group was dominated by individuals (>75%) at increased risk of post-traumatic stress disorder (age up to 30 years—79.63%; 31–40 years—78.95%; 41–50 years—88.46%; over 50 years—81.08%). However, the Pearson chi-square test showed no statistical relationship (Chi^2^ = 2.937, *p* = 0.401) between the age of the participants and the PDI cut-off point. Moreover, in terms of work experience and a score of ≥14 points on PDI, the vast majority of respondents (>80%) in each group were at increased risk of PTSD (up to 5 years of service—80.60%; 6–15 years 76.81%; 16–25 years 90.74%; over 25 years of service—83.64%). Analysis with Pearson chi-square test showed no statistical relationship (Chi^2^ = 4.293, *p* = 0.231) of work experience in relation to reaching the cut-off point.

Further analyses included any need for therapeutic intervention in relation to the respondents’ PDI scores (Table 4), as recommended by Guardia et al., 2013 [35]. A total of 6.12% of participants did not require intervention (<7 PDI score); 74.28% of respondents required further follow-up for PTSD and reassessment of PDI approximately 6 weeks after the previous survey; and 19.59% required steps concerning PTSD prevention and mitigation (>28 PDI score). Pearson’s chi-square test analysis showed that there was statistical significance in the relationship between gender and the level of PDI requiring therapeutic action (Chi2 = 12.507, *p* = 0.002), Pearson’s chi-square test analysis showed that there was statical significance in the relationship between gender and the level of PDI requiring therapeutic action (Chi^2^ = 12.507, *p* = 0.002), in both groups, the highest number of subjects were those who required further monitoring for PTSD (PDI scores of 7–28 were obtained by 76.72% of women and 66.07% of men). Similarly, relevance was shown for the age of respondents (Chi^2^ = 13.01, *p* = 0.043), where the majority (>59%) required follow-up for PTSD (age up to 30 years—66.67%; 31–40 years—80.26%; 41–50 years—80.77%; over 50 years—59.46%). There was also evidence of statistical significance (Chi^2^ = 11.110, *p* = 0.025) in terms of occupation and type of intervention. In all groups (>69%) there was a need for further follow-up for PTSD warnings (nurses—75.30%; paramedics—69.57%; other occupations—75.76%). In all job tenure groups, most people (>70%) achieved a score (7–28 on the PDI) qualifying them for further follow-up (up to 5 years of service—70.15%; 6–15 years—75.36%; 16–25 years—81.48%; over 25 years of service—70.91%). However, there was no statistical significance found in this respect (Chi^2^ = 4.649, *p* = 0.590).

## 4. Discussion

The COVID-19 pandemic, despite the fact it does not constitute a typical factor that can be identified as a direct cause of PTSD, has placed a significant traumatic burden on health professionals. There is a growing trend of anxiety and stress triggers that can lead to the development of PTSD among healthcare professionals as a consequence of experiencing the COVID-19 pandemic [30,36,37]. A review of studies confirms that healthcare professionals worldwide experienced psychological strain during the COVID-19 pandemic and, consequently, an increased risk of PTSD (Kang et al., 2020 [38]; Chen et al., 2020 [39]; Chidiebere Okechukwu et al., 2020 [40]; Shahrour and Dardas, 2020 [41]; Greenberg et al., 2020 [42]; Lamb et al., 2020 [43]). A review of the literature presents the high risk of PTSD among medical aid personnel during the COVID-19 pandemic, particularly affecting the North American region (Norman et al., 2020 [44]; Sagherian et al., 2020 [45]; Ayotte et al., 2020 [46]; Rodriguez et al., 2020 [47]; Shechter et al., 2020 [48]; Mehta et al., 2020 [49]; Crowe et al., 2020 [50]). Similarly, the analysis of studies also portrays a high level of exposure to PTSD among healthcare professionals in Europe (Vlah Tomičević and Lang, 2020 [51]; Alonso et al., 2020 [52]; Marco et al., 2020 [53]; Martínez-Caballeroi et al., 2020 [54]; Blanco-Daza et al., 2020 [55]; Luceño-Moreno et al., 2020 [56], Steudte-Schmiedgen et al., 2020 [57]), with a particular focus on the high risks in the Italian region: (Di Tella et al., 2020 [58]; Bassi et al., 2020 [59]; Marcomini et al., 2020 [60], Lasalvia et al., 2020 [61]). Polish studies also confirm that caring for patients during the COVID-19 pandemic resulted in an increased risk of PTSD (Nowicki et al., 2020 [62]; Kosydar-Bochenek et al., 2021 [63]). In the Asian region, the findings varied widely from an extremely low 2.3% of healthcare professionals at risk of developing PTSD in the study by Chinvararak et al., 2021 [64] to an extremely high 54.6% in the study by Jiang et al., 2020 [65].

The authors’ research has demonstrated that the risk level for PTSD among the population surveyed (N = 245) was very high, 82.44%. Similarly, Mirzaei et al., 2020 [66], and Kabunga and Okalo, 2021 [67] also obtained a high risk for PTSD amongst their respondents, 86% and 65.7% respectively. The average score in our study was 21.24 ± 8.97 PDI, similar results were obtained in studies conducted in Korea—Yoon et al., 2021 [30], PDI: 19.75 ± 8.82—and Italy—Carmassi et al., 2020 [32], PDI: 19.11 ± 8.29—which indicates a similar level of exposure to experiencing PTSD among the Polish population of healthcare professionals as in the Italian and Korean populations.

The conducted study uncovered a relationship between the sociodemographic characteristics and the risk of post-traumatic stress disorder. In terms of gender, females were shown to have a higher exposure to PTSD with a rate of 88.89%. Similar conclusions based on the study were shown by Blekas et al., 2020 [68]; Di Tella et al., 2020 [58]; Işik et al., 2020 [69] and Steudte-Schmiedgen et al., 2020 [57]. Similar conclusions were presented in Polish research by Kosydar-Bochenek et al., 2021 [63]; Bidzan et al., 2020 [70] and Rachubińska et al., 2022 [71]. However, Qutishat et al., 2020 [72], in a study conducted among Jordanian nurses, and Alanazi et al., 2020 [73], in a literature review concerning emergency healthcare professionals during the COVID-19 pandemic, showed that men were characterized by a higher incidence of PTSD. In contrast, no association between gender and the occurrence of increased risk of PTSD was found in studies by Zhou et al., 2020 [74] and Blanco-Daza et al., 2020 [55].

The authors’ research also presented a correlation between occupation and the risk of post-traumatic stress disorder among healthcare professionals, demonstrating that nurses are an occupational group particularly vulnerable to PTSD after experiencing trauma related to the COVID-19 pandemic. The above relationship was also found by Bulut et al., 2020 [75]; Geng et al., 2020 [76]; Shechter et al., 2020 [48]; and Song et al., 2020 [77], indicating that nurses are the most-burdened occupational group in terms of the occurrence of PTSD. A study by Lasalvia et al., 2020 [61] amongst 2195 Italian healthcare professionals concludes that being a nurse at least doubles the risk of developing posttraumatic stress symptoms. Research conducted in Poland by Szwamel et al., 2022 [78]; Haor et al., 2023 [79]; and Dymecka et al., 2022 [80] indicated that nurses were the group most exposed to stress in the face of the COVID-19 pandemic. This is mainly due to close contact with COVID-19 patients, the risk of infection, and overwork. In contrast, a different conclusion was drawn by Martínez-Caballero et al. on the basis of their study, 2020 [54], indicating a higher trauma burden for paramedics than for nurses. In contrast, Bahadirli and Sagaltici, 2020/2021 [81] conclude that the risk of PTSD is the highest in the group of doctors. Das et al., 2020 [82] presented no association between occupation and the risk of PTSD after experiencing trauma suffered during the COVID-19 pandemic.

Further, young age was enumerated as a risk factor for PTSD (Geng et al., 2020 [76]; Lamb et al., 2020 [43]; Shahrour and Dardas, 2020 [41]; Alonso et al., 2020 [52]; Chatzittofis et al., 2020 [83]). However, that was not confirmed in authors’ research. Other studies also showed that older people were at a greater risk of developing PTSD, such as Di Tella et al. 2020 [58].

The results of our study showed a partial correlation between the age of the respondents and the need for intervention in relation to PTSD risk. There is a relationship between the age and the PDI score, demanding further follow-up or immediate therapeutic support.

The current global research trends (Sanghera et al., 2019/2020 [84]; Luceño-Moreno et al., 2020 [56]; Nowicki et al., 2020 [62]) highlighted the tendency for a shorter length of service to influence the increased risk of PTSD after experiencing COVID-19-related trauma; however, the authors’ results showed no association with greater exposure to PTSD due to shorter work experience.

The trends shown in our research and global studies clearly show that the COVID-19 pandemic, although it had similar effects on the mental health of healthcare system employees, did not affect the world similarly. It certainly contributed to the risk burden of PTSD, but the strength of this risk varies across continents, regions, and countries. As shown in the US–Poland comparative study by Szaflarski, 2022 [85], US healthcare workers reported a stronger feeling of being overwhelmed by the COVID-19 pandemic. The geopolitical situation, cultural conditions, experiences of previous epidemic threats, or psychological support provided to healthcare system employees significantly affect the feeling of stress due to the COVID-19 pandemic and the risk of PTSD. In many studies, women and nurses are a group particularly vulnerable to PTSD as a result of the COVID-19 pandemic. It is difficult to determine whether these two characteristics are related, and which is dominant in the influence on the increased risk of PTSD. This is because the female gender predominates in the professional population of nurses. The analysis of the authors’ original research also does not give a clear answer to which trait is a predictor of PTSD. In the future, it would be required to conduct research on a representative sample of nurses in terms of a balanced gender division. Indeed, being a nurse and the resulting increased risk of PTSD in the COVID-19 pandemic can be explained by the tasks set for this professional group. The time of exposure to traumatic events in the case of nurses is also of great importance, because this professional group provided prolonged health services to patients with COVID-19. In Poland, nurses in many hospitals were on duty with patients, equipped with personal protective equipment, in a rotational system for several hours, and doctors carried out tasks depending on the needs of patients. The tasks of Polish nurses were based on nursing, continuous health monitoring, support, and the implementation of medical orders for patients, which resulted in heavy time, physical, and mental burden. Each occupational group carrying out tasks during the COVID-19 pandemic was significantly physically and mentally strained, which increased the risk of PTSD. The experience of trauma and the occurrence of PTSD symptoms for healthcare system employees is hazardous because, during this challenging time of the COVID-19 pandemic, they were expected to be fully dedicated, available, physically and mentally resilient, and fully professional in providing medical services. Awareness of this kind of responsibility further increases the risk of PTSD, preventing them from admitting their mental health weaknesses and actively seeking help.

## 5. Limitations

The authors’ study does not take into account all the possible correlations of the characteristics of the subjects with the occurrence of PTSD, nor does it answer the question of whether the next wave of the COVID-19 pandemic in Poland would cause a strong psychological burden on the healthcare system workers, causing long-term consequences in the form of PTSD. Therefore, it seems justified to continue systematic screening for mental stress among employees of the Polish healthcare system. Action must be taken to support those at risk of developing PTSD after experiencing trauma related to healthcare services. The issue of the incidence of PTSD as a result of trauma related to the COVID-19 pandemic is a rather recent topic, and requires further knowledge in this area, and the study presented in this thesis should be repeated in the near future.

In designing the study, it was assumed that the best research tool to assess the risk of PTSD in the long term after the trauma was the Peritraumatic Stress Scale, as opposed to the IES-R Event Impact Scale tool. The PDI has the advantage of being accessible in terms of understanding the questions posed to respondents, and the possibility of deepening the analysis of the results obtained in terms of taking action in response to the result obtained, increasing its sensitivity and reliability. However, the study is not without its limitations, inter alia, because of the risk of respondents referring to a distant past event unrelated to the COVID-19 pandemic, while distorting the overall average PDI scores. A limitation of this study is the way we reached study participants. The selection criterion may inadvertently overestimate the results obtained in the context of high exposure to PTSD by self-selecting for participation in the study. The form of data collection is also an important issue; Internet research has its challenges. The researchers are still determining whether a person participating in the study meets the assumed selection criteria. It is also not possible to observe the response of the examined person to the questions asked, which means that the answers given may not be valid. Another issue is the limited search range of target groups, due to the limited availability in Internet groups and forums. Due to numerous cyberattacks aimed at stealing data, trust in online research is severely limited. People are afraid to open unknown links from unknown addresses, due to the possibility of phishing, malware, ransomware, etc. In addition, the researcher can never be sure whether the person participating in the study is not trying to falsify the results by filling out the questionnaires many times by giving random answers.

## 6. Conclusions

The conducted study confirmed a high risk of post-traumatic stress disorder among healthcare professionals in Poland who experienced trauma related to the provision of healthcare during the COVID-19 pandemic. This risk is related to the gender of the subjects, with an indication of a higher risk of PTSD among women. The results have further shown a correlation between the increased risk of post-traumatic stress disorder and occupation, with nurses being the most affected group. The findings have demonstrated a partial relationship in relation to the age of the respondents, and the need for intervention in relation to the presence of PTSD hazards.

## Figures and Tables

**Table 1:** Sociodemographic analysis of the study group.

Variable	Category	Number (N)	Percentage (%)
Gender	Woman	189	77.14
	Man	56	22.86
Age	Up to 30 years of age	54	22.04
	31–40 years of age	76	31.02
	41–50 years of age	78	31.84
	over 50 years of age	37	15.10
Occupation	Nurse	166	67.76
	Paramedic	46	18.78
	Healthcare worker	4	1.63
	Medical Registrar	1	0.41
	Administrative Officer	7	2.86
	Physician	7	2.86
	Midwife	10	4.08
	Electroradiology technician	1	0.41
	Psychologist	2	0.82
	Sanitarian	1	0.41
Length of service	up to 5 years	67	27.35
	6–15 years	69	28.16
	16–25 years	54	22.04
	over 25 years	55	22.45

**Table 2:** Differences in PDI score in relation to the sociodemographic characteristics of the subjects.

Variable	Category	M	Me	SD	Statistical Analysis
Gender	Woman	22.52	23.00	8.18	Z = 3.873 *p* = 0.0001
	Man	16.91	16.50	10.19	
Age	Up to 30 years of age	21.76	20.50	10.74	F = 1.282 *p* = 0.281
	31–40 years of age	19.57	19.00	8.70	
	41–50 years of age	22.08	22.00	7.59	
	over 50 years of age	22.11	25.00	9.30	
Length of service	up to 5 years	21.88	21.00	10.27	F = 0.934 *p* = 0.425
	6–15 years	19.83	20.00	9.09	
	16–25 years	22.30	22.00	7.26	
	over 25 years	21.20	21.00	8.64	
Occupation	Nurse (I)	22.30	22.00	8.49	H = 6.998 *p* = 0.030 I > II
	Paramedic (II)	18.32	17.00	9.95	
	Other occupation (III)	19.97	21.00	9.13	

M—average, Me—median, SD—standard deviation, H—Kruskal–Wallis test, F—ANOVA analysis of variance, Z—Mann–Whitney test, *p*—statistical significance.

**Table 3:** Relationship of PDI cut-off score and sociodemographic characteristics of respondents.

Variable	Category	Cut-Off ≥14 Points PDI - PTSD Risk				Chi^2^ *p*
		≤13 pt		≥14 pt		
		N	%	N	%	
Gender	Woman	21	11.11	168	88.89	Chi^2^ = 23.698 *p* = 0.000
	Man	22	39.29	34	60.71	
Age	Up to 30 years of age	11	20.37	43	79.63	Chi^2^ = 2.937 *p* = 0.401
	31–40 years of age	16	21.05	60	78.95	
	41–50 years of age	9	11.54	69	88.46	
	over 50 years of age	7	18.92	30	81.08	
Length of service	Up to 5 years	13	19.40	54	80.60	Chi^2^ = 4.293 *p* = 0.231
	6–15 years	16	23.19	53	76.81	
	16–25 years	5	9.26	49	90.74	
	over 25 years	9	16.36	46	83.64	
Occupation	Nurse	20	12.05	146	87.95	Chi^2^ = 15.453 *p* = 0.001
	Paramedic	17	36.96	29	63.04	
	Other occupation	6	18.18	27	81.82	
Total		43	17.55	202	82.44	-

**Table 4:** Relationship between the need for intervention concerning PTSD and sociodemographic characteristics of respondents.

Variable	Category	PDI Result—Need for Intervention <7 pt. PDI—Lack of Intervention 7–28 PDI—Further Observation for PTSD >28 PDI—Urgent Intervention						Chi^2^ *p*
		<7 pt		7–28 pt		>28 pt		
		*n*	%	*n*	%	*n*	%	
Gender	Woman	6	3.17	145	76.62	38	20.11	Chi^2^ = 12.507 *p* = 0.002
	Man	9	16.07	37	66.07	10	17.86	
Age	Up to 30 years of age	5	9.26	36	66.67	13	24.07	Chi^2^ = 13.012 *p* = 0.043
	31–40	6	7.89	61	80.26	9	11.84	
	41–50	2	2.56	63	80.77	13	16.67	
	over 50 years of age	2	5.41	22	59.46	13	35.14	
Length of service	Up to 5 years	5	7.46	47	70.15	15	22.39	Chi^2^ = 4.649 *p* = 0.589
	6–15 years	6	8.70	52	75.36	11	15.94	
	16–25 years	1	1.85	44	81.48	9	16.67	
	over 25 years	3	5.45	39	70.91	13	23.64	
Occupation	Nurse	5	3.01	125	75.30	36	21.69	Chi^2^ = 11.110 *p* = 0.025
	Paramedic	5	10.87	32	69.57	9	19.57	
	Other occupation	5	15.15	25	75.76	3	9.09	
Total		15	6.12	182	74.28	48	19.59	-

## Data Availability

Data is available on request from the authors.
